# *In Vitro* Antiviral Activity and Resistance Profile of the Next-Generation Hepatitis C Virus NS3/4A Protease Inhibitor Glecaprevir

**DOI:** 10.1128/AAC.01620-17

**Published:** 2017-12-21

**Authors:** Teresa I. Ng, Rakesh Tripathi, Thomas Reisch, Liangjun Lu, Timothy Middleton, Todd A. Hopkins, Ron Pithawalla, Michelle Irvin, Tatyana Dekhtyar, Preethi Krishnan, Gretja Schnell, Jill Beyer, Keith F. McDaniel, Jun Ma, Guoqiang Wang, Li-Juan Jiang, Yat Sun Or, Dale Kempf, Tami Pilot-Matias, Christine Collins

**Affiliations:** a AbbVie, Inc., North Chicago, Illinois, USA; b Enanta Pharmaceuticals Inc., Watertown, Massachusetts, USA

**Keywords:** glecaprevir, NS3/4A protease inhibitor, HCV, antiviral activity, resistance, ABT-493

## Abstract

Glecaprevir (formerly ABT-493) is a novel hepatitis C virus (HCV) NS3/4A protease inhibitor (PI) with pangenotypic activity. It inhibited the enzymatic activity of purified NS3/4A proteases from HCV genotypes 1 to 6 *in vitro* (half-maximal [50%] inhibitory concentration = 3.5 to 11.3 nM) and the replication of stable HCV subgenomic replicons containing proteases from genotypes 1 to 6 (50% effective concentration [EC_50_] = 0.21 to 4.6 nM). Glecaprevir had a median EC_50_ of 0.30 nM (range, 0.05 to 3.8 nM) for HCV replicons containing proteases from 40 samples from patients infected with HCV genotypes 1 to 5. Importantly, glecaprevir was active against the protease from genotype 3, the most-difficult-to-treat HCV genotype, in both enzymatic and replicon assays demonstrating comparable activity against the other HCV genotypes. In drug-resistant colony selection studies, glecaprevir generally selected substitutions at NS3 amino acid position A156 in replicons containing proteases from genotypes 1a, 1b, 2a, 2b, 3a, and 4a and substitutions at position D/Q168 in replicons containing proteases from genotypes 3a, 5a, and 6a. Although the substitutions A156T and A156V in NS3 of genotype 1 reduced susceptibility to glecaprevir, replicons with these substitutions demonstrated a low replication efficiency *in vitro*. Glecaprevir is active against HCV with most of the common NS3 amino acid substitutions that are associated with reduced susceptibility to other currently approved HCV PIs, including those at positions 155 and 168. Combination of glecaprevir with HCV inhibitors with other mechanisms of action resulted in additive or synergistic antiviral activity. In summary, glecaprevir is a next-generation HCV PI with potent pangenotypic activity and a high barrier to the development of resistance.

## INTRODUCTION

Hepatitis C virus (HCV) is an enveloped, single-stranded, positive-sense RNA virus in the Flaviviridae family. Chronic HCV infection is a global health problem, with an estimated 80 million to 180 million people being infected worldwide (1, 2). If chronic HCV infection is not diagnosed or is left untreated, it can lead to serious liver diseases, such as cirrhosis, liver failure, and hepatocellular carcinoma. To date, seven distinct HCV genotypes, which differ in their geographic distributions, have been identified (1–3). Genotype 1 is the most prevalent genotype and accounts for approximately 45% of all HCV infections worldwide. Genotype 2 is more common in East and Southeast Asia, while genotype 3 is prevalent in Australia, South Asia, and a number of European countries. Genotype 4 is common in Egypt and the Middle East. Genotypes 5 and 6 are found primarily in South Africa and Southeast Asia, respectively, while genotype 7 has recently been identified in Central Africa (4).

The serine protease encoded by the HCV NS3 and NS4A genes is an attractive target for the discovery of direct-acting antivirals (DAAs). This protease is a viral enzyme responsible for cleaving the HCV polyprotein at four sites, yielding mature viral proteins essential for viral RNA replication (5). In addition to its crucial role in viral replication, HCV NS3/4A protease also plays a central role in the HCV innate immune evasion strategy by cleaving cellular proteins involved in the host innate antiviral response (6).

The first DAAs approved for use for the treatment of chronic HCV infection were inhibitors of HCV NS3/4A protease, namely, telaprevir and boceprevir, each of which is to be used in combination with pegylated interferon (pegIFN) and ribavirin (RBV) (7). Following these approvals in 2011, different interferon (IFN)-free DAA-containing regimens with or without an HCV NS3/4A protease inhibitor (PI) were approved for HCV therapy (7, 8). However, most of these approved DAAs are not equally potent across all HCV genotypes and subpopulations, nor do they consistently retain efficacy against HCV with specific substitutions associated with resistance to other members of the same inhibitor class (9–15). In addition, several currently approved regimens require different strategies to maximize efficacy, including the lengthening of the treatment duration (e.g., from 12 to 16 or 24 weeks) for certain populations or the addition of RBV, which in some patients could induce undesirable side effects (e.g., nausea, weight loss, or hemolytic anemia) (16–18). Lower efficacy has also been observed with a number of approved regimens in HCV-infected patients with baseline NS3 or NS5A amino acid polymorphisms that confer resistance to components of these regimens (19–24). Thus, there is an unmet medical need for a simple next-generation pegIFN- and RBV-free anti-HCV regimen with potent pangenotypic activity that can shorten treatment durations and provide high levels of efficacy in patients that are treatment naive or have previously failed a DAA-containing regimen.

**FIG 1:**
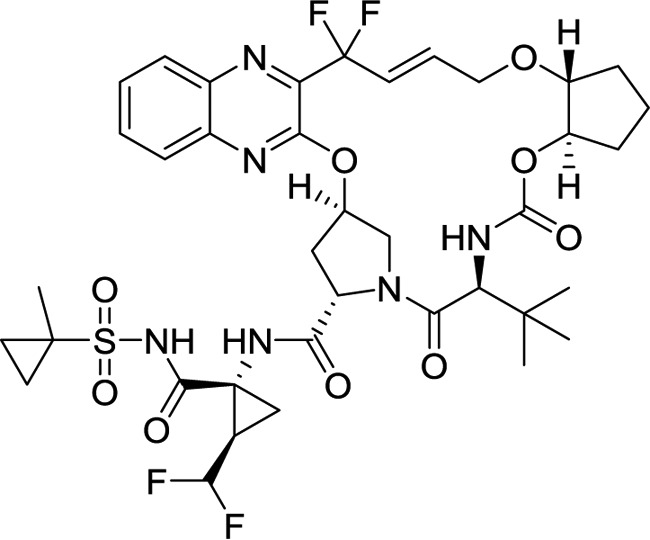
Chemical structure of glecaprevir.

Glecaprevir (formerly ABT-493; Fig. 1), a novel HCV NS3/4A PI with potent pangenotypic antiviral activity, is being developed for use in combination with the HCV NS5A inhibitor pibrentasvir (formerly ABT-530) for the treatment of HCV genotype 1 to 6 infection. Treatment with this combination regimen in treatment-naive or treatment-experienced (pegIFN, RBV, and/or sofosbuvir) patients infected with HCV genotypes 1 to 6 has resulted in a high sustained virologic response (SVR) rate, with <1% of patients experiencing virologic failure (25, 26). We report here the preclinical virologic characterization of glecaprevir, including characterization of its antiviral activity and resistance profile.

## RESULTS

### Activity of glecaprevir against HCV NS3/4A proteases and human proteases in biochemical assays.

Glecaprevir inhibited the enzymatic activity of HCV genotype 1 to 6 NS3/4A proteases, with the half-maximal (50%) inhibitory concentration (IC_50_) values ranging from 3.5 to 11.3 nM in a biochemical assay (Table 1). When glecaprevir was tested against six human serine proteases (chymase, chymotrypsin type II, chymotrypsin type VII, elastase, kallikrein, and urokinase) and one human cysteine protease (cathepsin B), no inhibition was observed at concentrations up to 200,000 nM. These results indicate that glecaprevir demonstrates a high level of selectivity for the HCV NS3/4A protease over the human proteases tested.

**TABLE 1:** Activity of glecaprevir against HCV NS3/4A proteases and human proteases in biochemical assays

Protease	HCV subtype or human protease^*a*^	Mean IC_50_ ± SD (nM)^*b*^
HCV	GT1a	4.6 ± 0.76
	GT1b	8.9 ± 1.6
	GT2a	3.5 ± 0.22
	GT2b	3.8 ± 0.96
	GT3a	7.9 ± 0.29
	GT4a	6.1 ± 1.9
	GT5a	8.1 ± 0.93
	GT6a	11.3 ± 1.8
Human	Chymase	>200,000
	Chymotrypsin type II	>200,000
	Chymotrypsin type VII	>200,000
	Elastase	>200,000
	Kallikrein	>200,000
	Urokinase	>200,000
	Cathepsin B	>200,000

a GT, genotype.

b Values were determined in ≥3 independent experiments. IC_50_, half-maximal (50%) inhibitory concentration.

### Antiviral activity and therapeutic index of glecaprevir *in vitro*.

Glecaprevir inhibited HCV subgenomic stable replicons containing proteases from HCV genotypes 1a, 1b, 2a, 2b, 3a, 4a, 5a, 6a, and 6e in Huh-7 cells, with 50% effective concentration (EC_50_) values ranging from 0.21 to 4.6 nM (Table 2). Compared with the activities of paritaprevir and grazoprevir, two recently approved HCV PIs, glecaprevir demonstrated improved activity against most of the replicons tested. Of note, glecaprevir was active against a replicon containing the protease from genotype 3, the most-difficult-to-treat HCV genotype, with an EC_50_ of 1.9 nM, which was 10- and 44-fold lower than the EC_50_s of paritaprevir and grazoprevir, respectively. To investigate the attenuation of the antiviral activity of glecaprevir due to its binding to plasma proteins when administered *in vivo*, the anti-HCV activity of glecaprevir was evaluated in the presence of 40% human plasma; there was a 6- to 11-fold decrease in the activity of glecaprevir against genotype 1a and genotype 1b replicon cells. Glecaprevir demonstrated no antiviral activity against human immunodeficiency virus type 1 (HIV-1) or hepatitis B virus (HBV) *in vitro* (HIV-1 EC_50_ = >22,000 nM, HBV EC_50_ = >32,000 nM). The cytotoxicity (expressed as the 50% cytotoxic concentration [CC_50_]) of glecaprevir was 72,000 nM (60 μg/ml) for Huh-7 cells harboring the genotype 1a replicon, resulting in an *in vitro* therapeutic index of 85,000-fold. The cytotoxicity of glecaprevir for cells of two additional cell lines was determined: HepG2 human liver cell line (CC_50_ = 62,000 nM, therapeutic index = 73,000-fold) and MT4 human lymphoid T cell line (CC_50_ = 59,000 nM, therapeutic index = 69,000-fold).

**TABLE 2:** Antiviral activity of glecaprevir and other HCV PIs *in vitro*^*a*^

HCV replicon or virus	Mean EC_50_ ± SD (nM)^*b*^		
	Glecaprevir	Paritaprevir	Grazoprevir
HCV stable replicons in 0% human plasma^*c*^			
GT1a H77	0.85 ± 0.15	1.0 ± 0.33	0.37 ± 0.05
GT1b Con1	0.94 ± 0.35	0.21 ± 0.07	1.0 ± 0.17
GT2a JFH-1	2.2 ± 1.1	9.8 ± 1.5	8.6 ± 3.0
GT2b	4.6 ± 1.2	107 ± 17	15 ± 2.0
GT3a	1.9 ± 0.62	19 ± 5.2	83 ± 18
GT4a	2.8 ± 0.41	0.09 ± 0.03	1.2 ± 0.31
GT5a SA13	1.4 ± 0.26	5.9 ± 1.6	2.1 ± 0.58
GT6a	0.86 ± 0.11	0.69 ± 0.09	0.89 ± 0.32
GT6e	0.21 ± 0.05	0.44 ± 0.17	0.25 ± 0.01
HCV stable replicons in 40% human plasma^*c*^			
GT1a H77	5.3 ± 1.0		
GT1b Con1	10 ± 5.0		
HIV-1	>22,000		
HBV	>32,000		

a PIs, protease inhibitors.

b Values were determined in ≥3 independent experiments. EC_50_, 50% effective concentration.

c Both the 0% and 40% human plasma assays also contained 5% fetal bovine serum. GT, genotype.

To characterize the activity of glecaprevir against HCV proteases from HCV-infected patients, the phenotypic susceptibilities to glecaprevir of a panel of replicons containing HCV proteases from a total of 40 clinical samples from patients infected with HCV genotypes 1 to 5 were determined in transient replicon assays. The median glecaprevir EC_50_s against replicons containing these clinical samples from patients infected with genotype 1a, 1b, 2a, 2b, 3a, 4a, 4d, and 5a were 0.08, 0.29, 1.6, 2.2, 2.3, 0.41, 0.17, and 0.12 nM, respectively, with an overall median EC_50_ of 0.30 nM (range, 0.05 to 3.8 nM) (Table 3). The baseline polymorphisms in this panel of clinical samples were assessed by comparison of their sequences with subtype-specific NS3 reference sequences. Five out of 11 genotype 1a clinical samples contained the NS3 polymorphism Q80K, and one sample had Q80N. No NS3 amino acid polymorphisms were detected in any of the clinical samples from patients infected with HCV genotype 1b, 2a, 2b, 3a, 4d, or 5a at amino acid positions associated with resistance to PIs.

**TABLE 3:** Antiviral activity of glecaprevir against HCV replicon cells containing HCV proteases from HCV-infected patients

HCV subtype	NS3 amino acid polymorphism^*a*^	No. of samples	EC_50_ (nM)^*b*^		
			Median	IQR^*c*^	Range
1a	None^*d*^	5	0.06	0.03	0.05–0.12
1a	Q80K	5	0.09	0.02	0.07–0.10
1a	Q80N	1	0.09		
1b	None	9	0.29	0.20	0.20–0.68
2a	None	4	1.6	0.42	0.66–1.9
2b	None	4	2.2	1.0	1.4–3.2
3a	None	2	2.3	1.6	0.71–3.8
4a	None	5	0.39	0.15	0.31–0.55
4a	T54S	1	0.44		
4d	None	3	0.17	0.06	0.13–0.25
5a	None	1	0.12		

a NS3 amino acid polymorphisms were assessed at amino acid positions 36, 43, 54, 55, 56, 80, 122, 155, 156, and 168 relative to the respective subtype-specific reference sequence.

b Values were determined in ≥3 independent transient-transfection assays.

c IQR, interquartile range.

d None, there were no polymorphisms relative to the subtype-specific reference sequence at the amino acid positions assessed.

### Characterization of amino acid substitutions selected by glecaprevir *in vitro*.

HCV subgenomic replicon cells containing the protease from genotype 1a, 1b, 2a, 2b, 3a, 4a, 5a, or 6a were passaged in the presence of glecaprevir at concentrations 10- or 100-fold its EC_50_s for the respective replicon cell lines to select for colonies with substitutions that conferred resistance to glecaprevir. Glecaprevir generally selected a low number of colonies containing resistance-conferring substitutions in replicon cells across the different HCV genotypes (Table 4). Higher colony counts were observed with selections using replicons with the genotype 2a JFH-1 backbone (i.e., the genotype 2a and 2b replicons in this study), regardless of the selection concentrations. This was likely due to the exceptionally high replication rates for replicons with this backbone (27).

**TABLE 4:** Selection of NS3 amino acid substitutions by glecaprevir in replicon cells containing proteases from HCV genotypes 1 to 6

HCV subtype^*a*^	Colony survival (%)^*b*^		NS3 amino acid substitution(s)	Prevalence in replicon selection^*c*^		Fold change in EC_50_^*d*^	Replication efficiency^*e*^ (%)
	10× EC_50_	100× EC_50_		10× EC_50_	100× EC_50_		
1a	0.043	0.0029	Q41R	5^*h*^/28		1.6	36
			A156T	2/28	9^*h*^/17	1,361	5.2
			A156V^*g*^		3/17	NV^*l*^	<0.5
			Q41R + I170V	7^*h*^/28		NV	<0.5
			V71A + I170V	3/28		3.3	1.0
			Q89R + A156T	1/28	5^*h*^/17	3,585	1.0
			Q89R + D168A	3/28			
1b	0.047	0.03	A156T	4^*h*^/25	3^*h*^/25	640	19
			A156V	9^*h*^/25	9^*h*^/25	1,786	9.2
			P89L + A156T	1/25	2/25	1,674	113
			P89L + A156V	8^*h*^/25	6^*h*^/25	4,243	119
			A156S + D168V	3/25			
			A156V + D168V		5/25	5,244	17
2a^*f*^	>0.05	>0.05	A156T	15^*h*^/24	5/23	216	
			A156V	9^*h*^/24	18^*h*^/23	1,143	
2b^*f*^	>0.05	>0.05	A156T	21/23	16/20	148	
			A156V	2/23	4^*h*^/20	1,455	
3a	0.1	0.0003	A156G	—^*i*^	1/3^*j*^	1,654	
			Y56H + Q168R	—^*i*^	2/3^*j*^	1,387	
4a	0.0015	0.0018	A156T	27^*h*^/36	8^*h*^/9	1,436	
			A156V	9/36	1/9	3,106	
5a	0.001	0	D168H	4/11	NA^*k*^	38	
6a	0.018	0	D168G	3^*h*^/25	NA	NV	
			D168H	10/25	NA	146	
			D168V	7^*h*^/25	NA	38	
			D168H + M179T	3/25	NA	153	

a The HCV subtype of the protease in the replicon cell lines.

b The data were calculated as follows: (number of surviving colonies/number of input replicon cells) × 100. The number of input replicon cells was 1 × 10^6^ cells for all selections except the genotype 3a 10× EC_50_ selection, for which the number of input replicon cells was 1 × 10^4^ cells.

c Unless indicated otherwise, the data represent the number of times that the amino acid substitution(s) was detected/total number of colonies analyzed.

d Relative to the glecaprevir EC_50_s for the respective wild-type replicons in transient-transfection assays, as follows: genotype 1a, 0.21 nM; genotype 1b, 0.47 nM; genotype 2a, 2.5 nM; genotype 2b, 3.1 nM; genotype 3a, 0.55 nM; genotype 4a, 0.67 nM; genotype 5a, 0.096 nM; and genotype 6a, 0.15 nM. Values were determined in ≥3 independent experiments.

e Relative to the replication efficiency of the wild-type replicon of the same subtype (which was given a value of 100%).

f The genotype 2a JFH-1 nonchimeric replicon was used for genotype 2a selection, and a genotype 2a JFH-1 chimeric replicon with the genotype 2b protease was used for genotype 2b selection.

g Substitutions detected in at least 1 patient who experienced virologic failure with the combination treatment of glecaprevir and pibrentasvir in phase 2 or 3 clinical studies are underlined.

h Number of times that the amino acid substitution(s) was detected by itself or in combination with other substitution(s).

i None of the substitutions detected in the colonies were found in >2 colonies.

j Only 3 colonies survived the selection.

k NA, not applicable.

l NV, not available, as the EC_50_ could not be determined due to the low replication efficiency of the replicon containing the amino acid substitution(s).

Glecaprevir selected substitutions at NS3 amino acid position A156 in replicons containing proteases from genotypes 1a, 1b, 2a, 2b, 3a, and 4a; replicons engineered with each of these A156 substitutions, if viable, demonstrated reduced susceptibility to glecaprevir by 148- to 3,106-fold (Table 4). Although the A156T and A156V substitutions in genotype 1 conferred high levels of resistance to glecaprevir, the viral fitness of replicons with these substitutions was low, with the replication efficiency being ≤5% or 19% for replicon cells containing the genotype 1a or genotype 1b protease with these substitutions, respectively. The viral fitness of replicons with these NS3 A156 substitutions in other HCV genotypes could not be accurately assessed because each of these NS3 proteins was expressed in the context of a chimeric replicon or with the replicon backbone of genotype 2a JFH-1. In replicon cells containing the genotype 3a protease, glecaprevir selected either A156G or Y56H in combination with Q168R, which conferred a 1,654- or 1,387-fold loss in susceptibility to glecaprevir, respectively. Glecaprevir selected substitutions at amino acid position D168 in replicon cells containing the genotype 5a or 6a protease, which reduced the susceptibility to glecaprevir by 38- to 146-fold.

In colonies of genotype 1 replicon cells selected by glecaprevir, A156 substitutions were occasionally detected in the presence of Q/P89 substitutions (Table 4). Replicons with substitutions at both positions Q/P89 and A156 were approximately 1.5- to 3-fold less susceptible to glecaprevir than those with the corresponding A156 substitution alone. The P89L substitution in genotype 1b appeared to increase the fitness of the A156 substitution-containing replicon *in vitro* (Table 4), but its role, if any, in the development of resistance to glecaprevir *in vivo* is not clear because the baseline prevalence of genotype 1b P89L was very low (2/1,583 patients) (unpublished data) and no PI-naive genotype 1b patients (*n* = 466) experienced virologic failure in phase 2/3 studies with the combination of glecaprevir and pibrentasvir (25, 26).

### Activity of glecaprevir and other HCV PIs against HCV replicons containing NS3 resistance-associated substitutions.

The activity of glecaprevir, paritaprevir, and grazoprevir against HCV genotype 1 to 6 replicons containing NS3 substitutions known to be associated with reduced susceptibility to approved PIs was evaluated (Table 5). Most of the common NS3 substitutions that confer resistance to approved PIs, including boceprevir, telaprevir, simeprevir, asunaprevir, and paritaprevir, are at amino acid position 155 or 168, whereas grazoprevir and voxilaprevir generally have lower levels of activity against replicons with various substitutions at NS3 amino acid position 156 or 168 (24, 28–32).

**TABLE 5:** Antiviral activity of glecaprevir and other HCV PIs against HCV replicons containing NS3 resistance-associated substitutions

HCV subtype	NS3 amino acid substitution	Glecaprevir		Paritaprevir		Grazoprevir	
		Mean EC_50_ ± SD (nM)^*a*^	Fold change^*b*^	Mean EC_50_ ± SD (nM)	Fold change	Mean EC_50_ ± SD (nM)	Fold change
1a	Wild type	0.21 ± 0.08		1.4 ± 0.4		0.14 ± 0.06	
	V36M	0.28 ± 0.10	1.4	3.0 ± 0.3	2.1	0.27 ± 0.06	1.9
	F43L	0.05 ± 0.01	0.3	27 ± 12	20	0.19 ± 0.05	1.4
	T54S	0.20 ± 0.06	1.0	0.54 ± 0.01	0.4	ND^*c*^	
	V55I	0.05 ± 0.01	0.2	1.4 ± 0.14	1.0	0.10 ± 0.02	0.7
	Y56H	0.21 ± 0.06	1.0	4.1 ± 1.1	2.9	2.2 ± 0.73	16
	Q80K	0.19 ± 0.05	0.9	3.9 ± 0.3	2.8	0.25 ± 0.09	1.8
	R155K	0.11 ± 0.03	0.5	51 ± 8.0	37	0.59 ± 0.19	4.2
	A156T	286 ± 93	1,361	24 ± 3.0	17	92 ± 6.0	655
	A156V	NV^*d*^		NV		NV	
	D168A	0.84 ± 0.45	4.0	70 ± 9.0	50	21 ± 9.8	154
	D168E	0.27 ± 0.09	1.3	20 ± 0.7	14	4.6 ± 0.66	33
	D168V	0.93 ± 0.28	4.4	135 ± 21	96	30 ± 13	211
	I170T	0.10 ± 0.02	0.5	ND		ND	
1b	Wild type	0.47 ± 0.13		0.11 ± 0.05		0.19 ± 0.05	
	T54A	0.45 ± 0.10	1.0	0.09 ± 0.01	0.8	ND	
	V55A	0.21 ± 0.03	0.4	0.07 ± 0.001	0.6	ND	
	R155K	0.27 ± 0.11	0.6	4.4 ± 0.1	40	0.21 ± 0.03	1.1
	A156T	301 **±** 62	640	0.81 ± 0.13	7.4	39 ± 4.3	203
	A156V	839 **±** 181	1,786	2.3 ± 0.53	21	68 ± 8.2	357
	D168A	0.69 ± 0.11	1.5	3.0 ± 0.2	27	8.5 ± 0.37	45
	D168E	0.40 ± 0.08	0.9	0.48 ± 0.05	4.4	0.78 ± 0.05	4.1
	D168V	1.5 ± 0.43	3.2	17 ± 2.0	159	4.1 ± 0.11	22
	V170A	0.49 ± 0.11	1.1	0.09 ± 0.03	0.8	ND	
2a	Wild type	2.5 ± 0.69		17 ± 2.0		10.5 ± 1.4	
	A156T	541 ± 77	216	29 ± 2.8	1.7	1,151 ± 144	110
	A156V	2,857 ± 235	1,143	37 ± 1.2	2.2	1,961 ± 244	187
	D168A	4.8 ± 1.3	1.9	306 ± 34	18	139 ± 9.4	13
	D168E	8.1 ± 1.9	3.3	89 ± 15	5.3	62 ± 10	5.9
	D168V	4.9 ± 0.79	2.0	228 ± 32	13	24 ± 0.94	2.3
2b	Wild type	3.1 ± 0.46		114 ± 24		6.1 ± 1.3	
	A156T	460 ± 172	148	338 ± 86	3.0	1,559 ± 925	256
	A156V	4,510 ± 1,726	1,455	573 ± 204	5.0	2,886 ± 482	473
	D168A	3.9 ± 1.0	1.3	1,309 ± 276	11	347 ± 113	57
	D168E	6.6 ± 1.8	2.1	256 ± 58	2.2	35 ± 6.0	5.7
	D168V	9.1 ± 1.2	2.9	1,073 ± 174	9.4	88 ± 19	14
3a	Wild type	0.55 ± 0.17		31 ± 7.0		47 ± 14	
	R155K	0.28 ± 0.03	0.5	161 ± 20	5.2	5.8 ± 0.64	0.1
	A156G	909 ± 349	1,654	1,768 ± 290	57	614 ± 148	13
	Q168R	30 ± 10	54	912 ± 221	29	165 ± 46	3.5
4a	Wild type	0.67 ± 0.23		0.048 ± 0.01		0.28 ± 0.04	
	R155C	1.7 ± 0.47	2.6	2.8 ± 0.25	59	2.0 ± 0.27	7.1
	A156T	962 ± 374	1,436	1.9 ± 0.25	40	296 ± 22	1,057
	A156V	2,081 ± 817	3,106	7.4 ± 1.6	155	169 ± 27	602
	D168H	15 ± 6.1	22	12 ± 3.0	252	25 ± 6.3	90
	D168V	6.5 ± 3.0	9.7	16 ± 1.9	323	13 ± 1.1	46
4d	Wild type	0.15 ± 0.04		0.015 ± 0.001		0.18 ± 0.01	
	D168V	0.28 ± 0.12	1.9	4.7 ± 0.91	312	2.5 ± 0.08	14
5a	Wild type	0.096 ± 0.03		0.33 ± 0.08		0.06 ± 0.02	
	D168E	0.41 ± 0.13	4.2	7.7 ± 1.7	23	0.58 ± 0.25	9.6
	D168H	3.6 ± 1.1	38	184 ± 65	558	15 ± 2.0	244
6a	Wild type	0.15 ± 0.03		0.12 ± 0.04		0.10 ± 0.02	
	D168A	12 ± 5.8	81	162 ± 80	1,347	103 ± 34	1,048
	D168H	22 ± 5.7	146	299 ± 87	2,490	45 ± 4.1	459
	D168V	5.8 ± 2.2	38	400 ± 139	3,336	24 ± 1.2	247

a Values were determined in ≥3 independent transient-transfection assays.

b Fold change relative to the glecaprevir EC_50_ for the respective wild-type replicon.

c ND, not determined.

d NV, not available, as the EC_50_ could not be determined due to the low replication efficiency of the replicon containing the amino acid substitution.

The NS3 Q80K substitution in genotype 1a, which reduces the efficacy of simeprevir in infected patients (22), did not confer resistance to glecaprevir, paritaprevir, or grazoprevir (Table 5). Other single amino acid substitutions at positions 36, 43, 54, 55, 56, and 170 in NS3 of genotype 1 that are associated with resistance to the PI class also had no impact on the antiviral activity of these three PIs (Table 5). None of the R155 substitutions in genotypes 1, 3, and 4 tested in this study reduced susceptibility to glecaprevir (≤2.6-fold decrease). The same substitutions slightly impacted susceptibility to grazoprevir (≤7.1-fold decrease) and conferred 5.2- to 59-fold reduced susceptibility to paritaprevir.

Glecaprevir retained activity (<5-fold increase in EC_50_) against all of the tested replicons with D168 substitutions in genotypes 1a, 1b, 2a, 2b, and 4d, while substitutions at position 168 in genotypes 3a, 4a, 5a, and 6a reduced susceptibility to glecaprevir by 4.2- to 146-fold (Tables 4 and 5). D168E, a common polymorphism found in genotype 5a NS3, reduced susceptibility to glecaprevir by only 4.2-fold (Table 5). Grazoprevir demonstrated 2.3- to 1,048-fold reduced activity against replicons with substitutions at D168 in genotypes 1a, 1b, 2a, 2b, 4a, 4d, 5a, and 6a. Although the Q168R substitution in genotype 3 conferred a low-level (3.5-fold) reduction in susceptibility to grazoprevir, the EC_50_ of grazoprevir against a replicon with this substitution (EC_50_ = 165 nM) was still higher than that of glecaprevir (EC_50_ = 30 nM) because the activity of grazoprevir against the wild-type genotype 3 replicon was low (EC_50_ = 47 nM). Almost all of the D/Q168 substitutions in genotypes 1 to 6 conferred higher levels of resistance to paritaprevir and grazoprevir than to glecaprevir.

Genotype 1 to 4 replicons containing A156 substitutions generally had low replication efficiencies, and not all of them were viable enough for drug susceptibility testing (Table 4). Both glecaprevir and grazoprevir demonstrated reduced activity against replicons with A156 substitutions in genotypes 1 to 4, whereas paritaprevir demonstrated reduced activity against replicons with these substitutions in genotypes 1, 3, and 4 but not genotype 2 (Table 5). The reduction in the antiviral activity against replicons with A156 substitutions in genotypes 1 to 4 was in the range of 148- to 3,106-fold for glecaprevir, 13- to 1,057-fold for grazoprevir, and 1.7- to 155-fold for paritaprevir. When tested against these 3 HCV PIs, the A156V substitution in genotypes 1 to 4 usually conferred a higher level of resistance than the A156T substitution in the corresponding genotypes.

### Activity of glecaprevir against HCV replicons containing amino acid substitutions that confer resistance to HCV NS5A inhibitors or NS5B polymerase inhibitors.

To determine the potential for the cross-resistance of glecaprevir with HCV DAAs with different mechanisms of action, glecaprevir was assessed in a transient-transfection assay with replicons containing key substitutions associated with resistance to either NS5A inhibitors or NS5B polymerase inhibitors (24, 33) (Table 6). Substitutions at NS5A amino acid positions M/L28, Q30, Y93, and P32 (deletion), as well as NS5B amino acid positions S282, C316, M414, Y448, S556, and S559, in genotype 1a or 1b replicon cells did not reduce susceptibility to glecaprevir. Thus, glecaprevir is active against HCV with key substitutions associated with resistance to these two classes of DAAs.

**TABLE 6:** Antiviral activity of glecaprevir against HCV replicons containing amino acid substitutions that confer resistance to NS5A inhibitors or NS5B polymerase inhibitors

DAA target	Genotype 1a		Genotype 1b	
	Amino acid substitution	Fold change in EC_50_^*a*^	Amino acid substitution	Fold change in EC_50_
NS5A	Wild type		Wild type	
	M28T	1.0	L28T	0.44
	M28V	1.1	P32 deletion	1.1
	Q30D	0.70	Y93H	1.4
	Q30R	1.3	Y93N	1.0
	Y93C	1.3		
	Y93H	1.4		
	Y93N	1.4		
NS5B	Wild type		Wild type	
	C316Y	1.1	S282T	0.74
	M414T	2.5	C316Y	0.85
	Y448C	1.6	Y448H	0.80
	Y448H	2.0	S556G	1.2
	S556G	1.4		
	S559G	2.2		

a Fold change relative to the glecaprevir EC_50_ for the respective wild-type replicon. Values were determined in ≥3 independent transient-transfection assays.

### Activity of glecaprevir in combination with HCV inhibitors with other mechanisms of action in HCV replicon cells.

The antiviral effect of the combination of glecaprevir with an HCV inhibitor with another mechanism of action was evaluated in a checkerboard combination assay using genotype 1b replicon cells. The HCV inhibitors tested with glecaprevir included the HCV NS5A inhibitor pibrentasvir (EC_50_ = 4.3 pM) (33), interferon alpha (IFN-α; EC_50_ = 1.2 IU/ml), and RBV (EC_50_ = 19 μM). Results from the checkerboard combination assay were analyzed using the MacSynergy II program (34). The combination of glecaprevir with pibrentasvir resulted in moderate synergistic antiviral activity, as reported previously (33), whereas the combination of glecaprevir with IFN-α or RBV resulted in additive antiviral activity (Table 7).

**TABLE 7:** Antiviral activity of the combination of glecaprevir with HCV inhibitors of other classes in HCV replicon cells

Drug combination^*a*^	Mean synergy vol ± SD (μM^2^ %)^*b*^	Mean antagonism vol ± SD (μM^2^ %)^*b*^	Interaction
Glecaprevir + IFN-α	15 ± 1.9	−1.1 ± 0.2	Additive
Glecaprevir + RBV	24 ± 4.9	−2.7 ± 0.2	Additive
Glecaprevir + pibrentasvir	73 ± 17	−1.9 ± 0.4	Moderate synergy

a IFN-α, interferon alpha; RBV, ribavirin. Pibrentasvir is an HCV NS5A inhibitor.

b Values were determined in ≥3 independent experiments.

**FIG 2:**
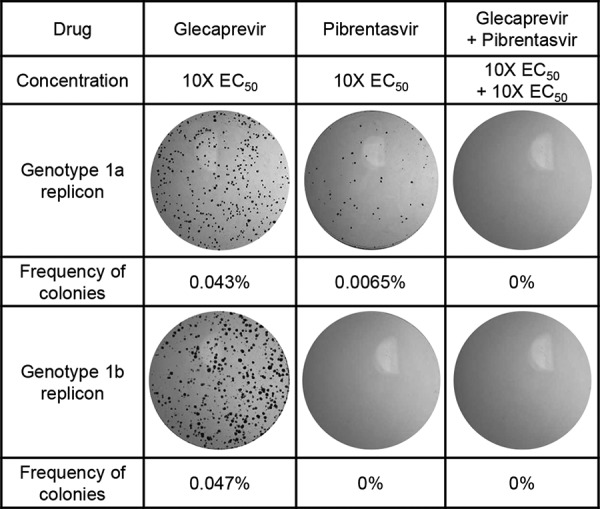
Inhibition of drug-resistant colony selection with the combination of glecaprevir and pibrentasvir in HCV genotype 1 replicon cells. Genotype 1a and 1b replicon cells seeded on 150-mm cell culture plates were treated for approximately 3 weeks with either glecaprevir or pibrentasvir individually or in combination at 10-fold the respective EC_50_ for each replicon cell line. All treatments also contained 400 μg/ml G418. Drug-resistant colonies that survived the treatment were fixed and stained with crystal violet, and the number of colonies was determined.

The combination of glecaprevir with pibrentasvir was further studied in a colony selection assay to determine the inhibition of the selection of colonies with substitutions conferring resistance to this combination. When cells containing the genotype 1a or genotype 1b replicon were treated with glecaprevir or pibrentasvir individually at 10-fold the respective EC_50_ for each replicon, a small percentage of replicon cells survived the drug pressure and formed colonies: 0.043% and 0.047% of genotype 1a and genotype 1b replicon cells survived glecaprevir treatment, respectively; 0.0065% of genotype 1a replicon cells and no genotype 1b replicon cells survived pibrentasvir treatment (Fig. 2). However, when either genotype 1a or 1b replicon cells were treated with a combination of glecaprevir and pibrentasvir, each at 10-fold the EC_50_ for the respective replicon, no replicon cells survived the selection, indicating that this combination resulted in enhanced suppression of the emergence of drug-resistant colonies in both replicon cell lines.

### Combination of glecaprevir with HIV-1 PIs *in vitro*.

The impact of two representative HIV-1 PIs, darunavir and lopinavir, on the anti-HCV activity of glecaprevir, as well as the impact of glecaprevir on the anti-HIV-1 activity of these two HIV-1 PIs, was also evaluated. The EC_50_ of glecaprevir for HCV genotype 1b Con1 replicon cells was 0.83 nM in this study (Table 8). This EC_50_ did not change in the presence of 100 nM darunavir or lopinavir (a concentration equivalent to approximately 5- to 10-fold the respective EC_50_ of each HIV-1 PI). Darunavir and lopinavir had EC_50_s of 10 and 23 nM, respectively, in the HIV-1 pNL4-3 antiviral assay. When these HIV-1 PIs were each tested in combination with 9.6 nM glecaprevir (a concentration equivalent to approximately 10-fold its EC_50_ for HCV genotype 1b Con1 replicon cells) in the HIV-1 assay, their respective EC_50_s remained the same as those when each HIV-1 PI was tested individually.

**TABLE 8:** Antiviral activity of glecaprevir and HIV-1 PIs alone or in combination in HCV replicon or HIV-1 infectivity assay

Antiviral assay	Compound(s)	Mean EC_50_ (nM)^*a*^
HCV genotype 1b Con1 replicon	Glecaprevir	0.83
	Glecaprevir + darunavir	0.78
	Glecaprevir + lopinavir	0.84
HIV-1 pNL4-3 infectivity	Darunavir	10
	Lopinavir	23
	Darunavir + glecaprevir	13
	Lopinavir + glecaprevir	22

a Values were determined in ≥2 independent experiments.

## DISCUSSION

Glecaprevir is a next-generation HCV NS3/4A PI with potent pangenotypic activity. In enzymatic assays, glecaprevir exhibited a high level of selectivity for HCV NS3/4A protease over human proteases. Glecaprevir exhibits an improved resistance profile in comparison with the other PIs currently approved for the treatment of HCV infections. Glecaprevir generally demonstrated a high genetic barrier to the development of resistance in drug-resistant colony selection assays with replicon cells containing proteases from different HCV genotypes. In addition, glecaprevir displayed synergistic antiviral activity when it was combined with the NS5A inhibitor pibrentasvir.

Among all HCV genotypes, genotype 3 infection is regarded as the most challenging to treat, and almost all currently approved PIs demonstrate reduced antiviral activity against HCV genotype 3 *in vitro* (9, 28, 30). Glecaprevir is a pangenotypic HCV PI that demonstrates similar inhibitory activity against HCV genotype 1 to 6 proteases in biochemical assays and HCV replication in replicon assays, with comparable activity against genotype 3a and the other genotypes. The clinical efficacy of the combination regimen of glecaprevir and pibrentasvir against HCV genotype 1 to 6 infection has been confirmed in different clinical studies, including >95% SVR rates in treatment-naive genotype 3-infected patients treated with the combination of glecaprevir and pibrentasvir for 8 weeks (35).

Glecaprevir demonstrates a resistance profile more favorable than those of other PIs currently approved for use for the treatment of HCV infections. As shown in Table 5, glecaprevir retained antiviral activity against HCV with most substitutions that are associated with resistance to the other approved HCV PIs, including those at NS3 amino acid position 80, 155, or 168 in different HCV genotypes. Glecaprevir is active against genotype 1 HCV with different D168 substitutions, most of which are known to confer resistance to paritaprevir and grazoprevir (28–30). Substitutions at NS3 amino acid position D/Q168 in genotypes 3, 5, and 6 were identified in resistance selection studies with glecaprevir and were found to reduce susceptibility to glecaprevir by approximately 38- to 146-fold. However, almost all of these D/Q168 substitutions conferred higher levels of resistance to paritaprevir and grazoprevir than glecaprevir. Although the A156G/T/V substitutions in genotype 1, 2, 3, or 4 confer substantial levels of resistance to glecaprevir and grazoprevir *in vitro*, HCV with these substitutions are predicted to have low viral fitness *in vivo*, on the basis of the low replication efficiency of genotype 1 replicons with the respective substitutions *in vitro* and the small number of drug-resistant replicon colonies containing these substitutions in the genotype 3 or 4 protease that survived selection with glecaprevir *in vitro*. On the basis of data compiled from 5,243 HCV-infected patients (36–40), the prevalence of glecaprevir resistance-conferring substitutions at amino acid position 156 or 168 was very low at baseline: A156T was detected in one (0.021%) genotype 1-infected patient, while none of the other glecaprevir resistance-conferring substitutions selected at these two amino acid positions *in vitro* were detected in any patients infected with genotype 1 (*n* = 4,795), 2 (*n* = 195), 3 (*n* = 78), 4 (*n* = 76), or 6 (*n* = 99) HCV.

If genotype 1 A156G/T/V substitutions emerged during DAA treatment in HCV-infected patients, they generally became undetectable within 3 months after virologic failure (41): A156G/T/V substitutions were detected at the failure time point in 16% (*n* = 14) of 85 genotype 1-infected patients who experienced virologic failure with a regimen containing grazoprevir in phase 2/3 studies. These substitutions did not persist in these patients, as only one of the 28 patients with available sequence information at the 12-week follow-up time point still had an A156G substitution. These observations regarding the low prevalence of genotype 1 NS3 A156G/T/V substitutions at the failure and follow-up time points are indicative of low viral fitness *in vivo* and are consistent with the low viral fitness of genotype 1 replicons containing the A156T/V substitutions *in vitro*.

As shown in Table 4, replicon cells containing the A156 substitutions in genotype 2a and 2b proteases appeared to be quite prevalent in a drug-resistant colony selection study with glecaprevir. However, among the 358 HCV genotype 2a- or 2b-infected patients treated with the combination regimen of glecaprevir and pibrentasvir in phase 2/3 studies, only two patients experienced virologic failure and A156 substitutions were not found to have emerged in either patient at the time of virologic failure (25, 42). These data support the prediction that genotype 2 HCV with A156 substitutions, like genotype 1 HCV with the same substitutions, may demonstrate low viral fitness *in vivo*. The high prevalence of replicon colonies with genotype 2a or 2b NS3 A156 substitutions observed with glecaprevir selection was likely due to the exceptionally high replication rate of genotype 2a JFH-1 (27), which constituted the backbone of both of the genotype 2a and 2b replicons in this study.

The potent *in vitro* anti-HCV activity of glecaprevir has translated into robust clinical efficacy. When glecaprevir was evaluated in a 3-day dose-ranging monotherapy study in HCV genotype 1-infected treatment-naive patients, the mean maximal decreases in the HCV plasma viral load from the baseline at the end of the 3-day monotherapy ranged from 3.8 to 4.3 log_10_ IU/ml for daily doses of 100 to 700 mg (43). Of note, NS3 amino acid substitutions emerged in only 2 (4%) of the 48 patients in this monotherapy study, with either T54S or A156T emerging in one patient each infected with HCV genotype 1a. Genotype 1a T54S does not confer resistance to glecaprevir, whereas A156T confers a 1,361-fold decrease in susceptibility to glecaprevir, but a replicon with this substitution has a replication efficiency of only 5% of that of the wild-type replicon. This low rate of emergence of amino acid substitutions during monotherapy is consistent with the high barrier to resistance observed for glecaprevir in genotype 1a replicons *in vitro*.

The combination of glecaprevir and pibrentasvir, two DAAs with distinct anti-HCV mechanisms of action, resulted in moderate synergistic inhibition of the replication of HCV replicons. The combination of these two DAAs with nonoverlapping resistance profiles also enhanced the suppression of the emergence of drug-resistant replicon colonies *in vitro*. In phase 2/3 studies, the combination of 300 mg glecaprevir and 120 mg pibrentasvir, dosed once daily, achieved an overall SVR rate of >99% (modified intent to treat [mITT]) in 2,256 patients infected with HCV genotypes 1 to 6 with a treatment duration of 8, 12, or 16 weeks, regardless of patient or viral characteristics (25, 26). These 2,256 patients included treatment-naive and treatment-experienced (pegIFN, RBV, and/or sofosbuvir) patients with compensated cirrhosis or without cirrhosis. An 8-week combination regimen with glecaprevir and pibrentasvir for treatment-naive patients without cirrhosis infected with HCV genotypes 1 to 6 reduces the treatment duration, as the usual duration of other approved anti-HCV therapies is 12 weeks. Among the 22 patients experiencing virologic failure with the combination regimen of glecaprevir and pibrentasvir in these studies, treatment-emergent NS3 substitutions at amino acid positions 56, 80, 156, and 168 were detected in 12 patients (25). Only three of these 12 patients had HCV with substitutions at NS3 amino acid position 156: one genotype 1a-infected patient and two genotype 3a-infected patients. These data demonstrate that the combination regimen of glecaprevir and pibrentasvir is highly efficacious in patients infected with HCV genotypes 1 to 6. In addition, although substitutions at NS3 amino acid position A156 are capable of significantly reducing the susceptibility to glecaprevir *in vitro*, these substitutions were rarely detected at the time of virologic failure in patients who experienced virologic failure.

The combination of glecaprevir and pibrentasvir was also evaluated in HCV genotype 1-infected patients who had failed a prior DAA-containing regimen (44). HCV samples collected from these patients at baseline had diverse resistance profiles and a high prevalence of NS3 and/or NS5A resistance-associated substitutions due to the patients' failure to their prior DAA treatments. This combination regimen achieved an SVR rate of 96% (mITT), demonstrating its robust efficacy in HCV-infected DAA-experienced patients.

In summary, consistent with its preclinical profile, glecaprevir has demonstrated clinical efficacy against all major HCV genotypes, including the most-difficult-to-treat genotype, genotype 3. Glecaprevir is a potent pangenotypic HCV DAA that maintains activity against HCV with the most common NS3 resistance-associated substitutions found in HCV-infected patients who had previously failed a regimen containing another approved HCV PI. To date, the combination regimen of glecaprevir and pibrentasvir has demonstrated robust clinical efficacy in patients infected with HCV genotypes 1 to 6, including patient populations with other medical conditions, such as HIV-1 coinfection, compensated cirrhosis, and chronic kidney disease (45–47).

## MATERIALS AND METHODS

### Compounds.

Glecaprevir [(3a*R*,7*S*,10*S*,12*R*,21*E*,24a*R*)-7-*tert*-butyl-*N*-[(1*R*,2*R*)-2-(difluoromethyl)-1-{[(1-methylcyclopropyl)sulfonyl] carbamoyl}cyclopropyl]-20,20-difluoro-5,8-dioxo-2,3,3a,5,6,7,8,11,12,20,23,24a-dodecahydro-1*H*,10*H*-9,12-methanocyclopenta[18,19][1,10,17,3,6]trioxadiazacyclononadecino[11,12-*b*]quinoxaline-10-carboxamide; identified by AbbVie and Enanta] (Fig. 1), pibrentasvir (33), paritaprevir (28), and lopinavir were synthesized at AbbVie. Grazoprevir (17) was purchased from ApexBio (Houston, TX), and darunavir, IFN-α, and RBV were purchased from Sigma-Aldrich (St. Louis, MO).

### Inhibition of HCV NS3/4A proteases by glecaprevir in a biochemical assay.

Eight recombinant HCV NS3/4A proteases were generated for use in evaluating glecaprevir activity in a biochemical assay. Each recombinant protein contained the entire coding regions of NS3 (amino acids 1 to 631) and NS4A (amino acids 1 to 54) from HCV genotypes 1 to 6, a 6-histidine tag at the N terminus to facilitate purification by affinity chromatography, and three lysine residues at the C terminus to increase the solubility of the protein. Genes encoding NS3/4A were derived from laboratory strains 1a-H77 (GenBank accession number NC_004102) and 1b-N (GenBank accession number AF139594) or from clinical samples from patients infected with genotype 2a, 2b, 3a, or 4a. All patients provided written informed consent. Clinical studies were designed according to Good Clinical Practice guidelines, the Declaration of Helsinki, and applicable local regulations, with independent ethics committee or institutional review board approval for all study sites. The genotype 5a NS3/4A gene sequence was synthetically constructed based on the sequence of the clinical isolate SA13 (GenBank accession number AF064490) (48), whereas the genotype 6a NS3/4A gene sequence was synthetically constructed based on a consensus sequence derived from the alignment of 15 genotype 6a sequences available in GenBank. The NS3/4A genes were each cloned into the protein expression vector pET14b (Novagen, Madison, WI), and a clone with an NS3/4A protease sequence that matched the consensus sequence for each sample was subsequently selected for protein expression and purification. Protease activity was measured by continuous monitoring of the fluorescence change associated with the cleavage of a fluorogenic depsipeptide (EDANS/DABCYL) substrate using a purified recombinant HCV NS3/4A protease as described previously (49). The IC_50_ for each HCV protease was determined in studies in which the protease was preincubated with glecaprevir for 30 min. The percent inhibition was calculated from the initial rates of the inhibited reactions relative to the rate for the uninhibited control.

### Inhibition of human proteases by glecaprevir in biochemical assays.

The activity of glecaprevir against seven human proteases (Sigma-Aldrich, St. Louis, MO) was evaluated in biochemical assays with a 20-min incubation at room temperature as follows: (i) for elastase, 50 mM Tris, pH 7.5, 100 mM NaCl, 0.1% Triton X-100, 0.25 mM substrate (methoxy [MeO]-succinyl [Suc]-AAPV-7-amino-4-methylcoumarin (AMC) ; catalog number M9771; Sigma-Aldrich), and 0.05 U/ml elastase (excitation wavelength = 370 nm, emission wavelength = 460 nm); (ii) for chymase, 0.4 M Tris, pH 8.0, 1.8 M NaCl, 9% dimethyl sulfoxide, 0.4 mM substrate (Suc-AAF-AMC; catalog number S8758; Sigma-Aldrich), and 0.063 U/ml chymase (excitation wavelength = 370 nm, emission wavelength = 460 nm); (iii) for cathepsin B, 50 mM MES (morpholineethanesulfonic acid), pH 6.0, 50 mM NaCl, 2 mM EDTA, 0.1% Triton X-100, 0.4 mM substrate (Z-RR-AMC; catalog number C5429; Sigma-Aldrich), and 1.7 μg/ml cathepsin B (excitation wavelength = 370 nm, emission wavelength = 460 nm); (iv and v) for chymotrypsin types II and VII, 50 mM Tris, pH 8, 10 mM CaCl_2_, 0.1% Triton X-100, 0.25 mM substrate (Suc-AAF-AMC; catalog number S8758; Sigma-Aldrich), and 0.5 nM chymotrypsin type II or type VII (excitation wavelength = 370 nm, emission wavelength = 460 nm); (vi) for kallikrein, 50 mM Tris, pH 7.4, 0.5 mM EDTA, 0.05% Tween 20, 150 mM NaCl, 0.35 mM substrate (H-Pro-Phe-Arg-AMC; catalog number I-1295; Bachem), and 2 nM kallikrein (excitation wavelength = 355 nm, emission wavelength = 460 nm); and (vii) for urokinase, 50 mM Tris, pH 7.4, 0.15 M NaCl, 0.5% Pluronic F-68, 0.2 mM substrate (pyro-Glu Arg-*p*-nitroanilide-HCl; catalog number S-2444; Diapharma), and 3 nM urokinase (absorbance at 405 nm).

### Antiviral activity of glecaprevir and other HCV PIs in stable HCV replicon cells.

The activity of glecaprevir, paritaprevir, or grazoprevir against cells of nine cell lines each stably transfected with an HCV subgenomic replicon containing NS3 protease from a different HCV genotype was determined using a luciferase reporter assay as described previously (28). Five of these nine cell lines have been described previously, including those transfected with genotypes 1a H77, 1b Con1, 3a, 4a, and 6a (28). The other four cell lines were established by transfecting cells with a nonchimeric genotype 2a JFH-1 replicon (GenBank accession number AB047639), two genotype 2a JFH-1 chimeric replicons containing either a genotype 2b NS3 protease domain (N-terminal 251 amino acids) or a sequence encoding full-length NS3 through the first 39 amino acids of NS5B from genotype 5a (strain SA13), and one chimeric replicon with a genotype 1b Con1 backbone containing full-length NS3 and NS4A sequences from genotype 6e. The genotype 2b and 6e NS3 sequences were each synthetically constructed based on a consensus sequence derived from the alignment of 15 genotype 2b and 4 genotype 6e sequences, respectively. All replicon constructs were bicistronic subgenomic replicons similar to those described by Bartenschlager and coworkers, and the replicon cell lines were generated by introducing these constructs into cells of an Huh-7 human hepatoma-derived cell line (50). The inhibitory effect of the PIs on HCV replication in replicon cells was determined in Dulbecco's modified Eagle medium containing 5% fetal bovine serum with or without 40% human plasma (Bioreclamation, Westbury, NY). The EC_50_s were determined using nonlinear regression curve fitting as described previously (33).

### Cytotoxicity of glecaprevir.

The cytotoxicity of glecaprevir for Huh-7, HepG2, and MT4 cells was determined by the 3-(4,5-dimethylthiazol-2-yl)-2,5-diphenyltetrazolium bromide (MTT; Sigma-Aldrich, St. Louis, MO) colorimetric assay. Huh-7 cells containing the genotype 1a replicon or HepG2 cells (4,000 cells/well) were plated in 96-well plates and incubated overnight before the addition of glecaprevir. For MT4 cells, 10,000 cells/well were plated immediately before the addition of glecaprevir. The rest of the assay was performed as described previously (33).

### Activity of glecaprevir against human immunodeficiency virus type 1 and hepatitis B virus.

The antiviral activity of glecaprevir against HIV-1 and HBV was determined at the Southern Research Institute as described previously (33). Briefly, glecaprevir was tested in an HIV-1 antiviral cytoprotection assay using CEM-SS cells and the IIIB strain of HIV-1 or was tested in an anti-HBV assay by directly measuring the extracellular HBV DNA copy number in a real-time quantitative PCR (TaqMan) assay.

### Antiviral activity of glecaprevir against HCV replicons containing protease genes from HCV-infected patients.

The activity of glecaprevir against HCV replicons containing protease genes from individual patients infected with HCV genotypes 1 to 5 was determined by testing the susceptibility of a panel of chimeric or nonchimeric replicons containing the respective NS3 or NS3/4A gene. Each of the NS3 gene sequences from genotypes 1, 2, and 4, with the exception of two genotype 2b sequences, was derived from serum or plasma from an HCV-infected patient by bulk PCR amplification, producing a population of sequences representing the viral quasispecies. Two genotype 2b sequences were synthesized based on the consensus sequences of the respective samples. One genotype 3a sequence was synthesized based on a consensus sequence from the sequences available in the Los Alamos HCV sequence database, and another genotype 3a sequence was a clonal sequence derived from a patient sample. The genotype 5a sequence (strain SA13) was synthesized based on the published sequence of the HCV isolate (48).

Transient replicons containing NS3 from clinical samples from HCV genotype 1a- and 1b-infected patients were described previously (28). For the transient replicons containing NS3/4A from samples from genotype 3a-infected patients, the first 251 amino acids of NS3 and the region encoding NS4A amino acids 21 to 32 (numbered relative to the NS4A coding region) from these HCV samples were cloned to replace the corresponding residues from the backbone of a genotype 1b Con1 shuttle vector. A genotype 1b Con1 transient replicon shuttle vector was also used to clone the region encoding the first 251 amino acids of NS3 from samples from genotype 4a- or 4d-infected patients in place of the corresponding region from HCV genotype 1b Con1.

A genotype 2a JFH-1 replicon shuttle vector was used to generate transient replicons containing NS3 from clinical samples from genotype 2a- and genotype 2b-infected patients. The region encoding the first 227 amino acids of NS3 from samples from genotype 2a- and 2b-infected patients was used to replace the corresponding region in the genotype 2a JFH-1 replicon shuttle vector. The genotype 2a JFH-1 replicon shuttle vector was also used to generate a transient replicon containing a region encoding full-length NS3 through the first 39 amino acids of NS5B from genotype 5a (strain SA13).

For the analysis of NS3 sequences from patients, we defined a polymorphism to be an amino acid difference in the sequence of a baseline sample relative to the sequence of the appropriate HCV subtype reference sequence: genotype 1a H77 (GenBank accession number NC_004102), genotype 1b Con1 (GenBank accession number AJ238799), genotype 2a JFH-1 (GenBank accession number AB047639), genotype 2b HC-J8 (GenBank accession number D10988), genotype 3a S52 (GenBank accession number GU814263), genotype 4a ED43 (GenBank accession number GU814265), genotype 4d QC382 (GenBank accession number FJ462437), and genotype 5a SA13 (GenBank accession number AF064490). Polymorphisms were assessed at the following NS3 amino acid positions that are associated with resistance to the HCV PI class: 36, 43, 54, 55, 56, 80, 122, 155, 156, and 168. All DNA sequencing was performed by the Sanger sequencing method.

### Selection of amino acid substitutions by glecaprevir in replicon cells.

To characterize the amino acid substitutions that confer reduced susceptibility to glecaprevir, resistance selection was conducted with stable HCV replicon cell lines containing genotype 1a, 1b, 2a, 2b, 3a, 4a, 5a, or 6a NS3 as described previously (28, 33). Briefly, 10^6^ replicon cells were plated in 150-mm cell culture plates and cultured in the presence of G418 (400 μg/ml) and glecaprevir at a concentration that was 10- or 100-fold above its EC_50_s for the respective replicon cell lines. After approximately 3 weeks of treatment, the surviving replicon colonies in one plate for each selection condition were stained with crystal violet and counted, whereas approximately 20 to 30 colonies in a replicate plate or most of the colonies, if less than 20 colonies survived the selection, were picked and further expanded for genotyping. The expanded colonies were lysed in CellsDirect lysis buffer (Invitrogen, MA), and the NS3-coding region from each colony was amplified by reverse transcription-PCR and sequenced. For genotype 1a, 1b, 2a, and 5a colonies, the full-length NS3 sequence was determined, whereas for colonies of the chimeric replicon containing the N-terminal region of NS3 from genotype 2b, 3a, 4a, or 6a, the sequence encoding the NS3 N-terminal region was determined. We defined a substitution in a replicon to be an amino acid difference in a sequence from a posttreatment sample relative to the sequence of the corresponding untreated sample. For resistance selection studies, NS3 substitutions detected at the following amino acid positions associated with resistance to the HCV PI class in >2 colonies in each replicon selection are reported: 36, 43, 54, 55, 56, 80, 122, 155, 156, and 168. In addition, substitutions detected at other amino acid positions in the NS3 protease domain in >2 colonies in each replicon selection are also reported. Selection with the genotype 3a replicon cell line at a concentration 100-fold above the EC_50_ resulted in only three colonies, and the substitutions in all three colonies are reported.

### Effects of amino acid substitutions on the antiviral activity of glecaprevir and other HCV PIs in HCV replicon cells.

Replicons containing the substitutions of interest in NS3 (genotypes 1a, 1b, 2a, 2b, 3a, 4a, 4d, 5a, and 6a) were constructed to evaluate their susceptibility to glecaprevir, paritaprevir, or grazoprevir in transient replicon assays as described previously (28). In addition, the activity of glecaprevir against replicons containing substitutions in NS5A or NS5B (genotypes 1a and 1b) was also tested (33). Mutagenesis was performed using a Change-IT multiple-mutation site-directed mutagenesis kit (USB, Cleveland, OH) or by cloning a synthesized DNA fragment encoding the amino acid substitution(s). The genotype 1a and 1b replicon shuttle vectors used for the introduction of the substitutions of interest in the NS3, NS5A, or NS5B gene for transient-transfection assays have been described previously (28, 33). The replicon shuttle vectors used for the introduction of substitutions in NS3 genes from other genotypes were similar to the stable replicon cell line constructs described above, except for the absence of the *neo* gene. After mutagenesis, the plasmids were linearized and RNA was transcribed and then used to transfect Huh-7 cells. Inhibition of HCV replication by the HCV inhibitors was measured by luciferase assay, as described above. Replication efficiency was calculated as a percentage of wild-type replication, as described previously (28).

### Combinations of glecaprevir with HCV inhibitors with other mechanisms of action in HCV replicon cells.

The antiviral effect produced by the combination of glecaprevir with an HCV inhibitor with a different mechanism of action was studied in genotype 1b Con1 replicon cells as described previously using the checkerboard method to determine if the combination produced synergistic, additive, or antagonistic effects (33). The resulting data were analyzed using the MacSynergy II program (34, 51, 52). The assignment of the synergy or antagonism volumes was based on the guidelines provided in the MacSynergy II program user's manual (34).

For the selection of drug-resistant HCV replicon colonies with the combination of glecaprevir and pibrentasvir, genotype 1a or 1b replicon cells were treated for approximately 3 weeks with either one or both inhibitors at a concentration that was 10-fold the EC_50_ for the respective replicon cell line, as described above. The drug-resistant colonies were fixed and stained with crystal violet, and the number of colonies was determined.

### Combination of glecaprevir with HIV-1 PIs in HIV-1 antiviral assays.

The anti-HIV-1 activity of an HIV-1 PI (darunavir or lopinavir) either alone or in combination with glecaprevir was studied in a 5-day cytoprotection assay using MT4 cells and the pNL4-3 strain of HIV-1. The study was designed to determine whether glecaprevir at a concentration 10-fold its EC_50_ for HCV genotype 1b Con1 replicon cells (EC_50_ = 0.96 nM) affected the antiviral activity of darunavir or lopinavir against HIV-1 in MT4 cells. Briefly, darunavir or lopinavir was serially diluted to 8 concentrations that covered the respective HIV-1 EC_50_ in the middle of the dose range. Each HIV-1 PI concentration, with or without the combination with 9.6 nM glecaprevir, was added to HIV-1-infected cells. The plates were incubated at 37°C for 5 days. After this period, 25 μl MTT reagent (4 mg/ml) was added to the cells, and the plates were incubated for 4 h. The MTT formazan formed in the cells was solubilized by 50 μl of acidified SDS overnight. The plates were read at wavelengths of 570 and 650 nm using a spectrophotometer. The EC_50_s of darunavir or lopinavir for HIV-1 in the presence or absence of glecaprevir were calculated as described above.

### Combination of glecaprevir with HIV-1 PIs in HCV replicon antiviral assays.

The anti-HCV activity of glecaprevir either alone or in combination with an HIV-1 PI (darunavir or lopinavir) was studied in HCV replicon cells. To this end, darunavir or lopinavir at 0.1 μM (a concentration equivalent to approximately 5- to 10-fold the respective HIV-1 EC_50_) was combined with six serial dilutions of glecaprevir to treat HCV genotype 1b Con1 replicon cells. The range of glecaprevir concentrations tested was chosen to ensure that its EC_50_ in genotype 1b Con1 replicon cells (0.96 nM) was in the middle of the serial dilution range. The inhibitory effects of glecaprevir either alone or in combination with one of these HIV-1 PIs on the replication of the HCV replicon were determined by measuring the luciferase reporter activity as described above.

## References

[1] Messina JP, Humphreys I, Flaxman A, Brown A, Cooke GS, Pybus OG, Barnes E 2015 Global distribution and prevalence of hepatitis C virus genotypes. Hepatology 61:77–87. doi:10.1002/hep.27259. 25069599 PMC4303918

[2] Gower E, Estes C, Blach S, Razavi-Shearer K, Razavi H 2014 Global epidemiology and genotype distribution of the hepatitis C virus infection. J Hepatol 61:S45–S57. doi:10.1016/j.jhep.2014.07.027. 25086286

[3] Smith DB, Bukh J, Kuiken C, Muerhoff AS, Rice CM, Stapleton JT, Simmonds P 2014 Expanded classification of hepatitis C virus into 7 genotypes and 67 subtypes: updated criteria and genotype assignment web resource. Hepatology 59:318–327. doi:10.1002/hep.26744. 24115039 PMC4063340

[4] Murphy DG, Sablon E, Chamberland J, Fournier E, Dandavino R, Tremblay CL 2015 Hepatitis C virus genotype 7, a new genotype originating from central Africa. J Clin Microbiol 53:967–972. doi:10.1128/JCM.02831-14. 25520447 PMC4390628

[5] Bartenschlager R, Ahlborn-Laake L, Mous J, Jacobsen H 1994 Kinetic and structural analyses of hepatitis C virus polyprotein processing. J Virol 68:5045–5055. 8035505 10.1128/jvi.68.8.5045-5055.1994 PMC236447

[6] Horner SM, Gale M Jr 2013 Regulation of hepatic innate immunity by hepatitis C virus. Nat Med 19:879–888. doi:10.1038/nm.3253. 23836238 PMC4251871

[7] Kwong AD 2014 The HCV revolution did not happen overnight. ACS Med Chem Lett 5:214–220. doi:10.1021/ml500070q. 24672647 PMC3963459

[8] Asselah T, Boyer N, Saadoun D, Martinot-Peignoux M, Marcellin P 2016 Direct-acting antivirals for the treatment of hepatitis C virus infection: optimizing current IFN-free treatment and future perspectives. Liver Int 36(Suppl 1):47–57. doi:10.1111/liv.13027. 26725897

[9] Gottwein JM, Scheel TK, Jensen TB, Ghanem L, Bukh J 2011 Differential efficacy of protease inhibitors against HCV genotypes 2a, 3a, 5a, and 6a NS3/4A protease recombinant viruses. Gastroenterology 141:1067–1079. doi:10.1053/j.gastro.2011.06.004. 21699793

[10] Romano KP, Ali A, Aydin C, Soumana D, Ozen A, Deveau LM, Silver C, Cao H, Newton A, Petropoulos CJ, Huang W, Schiffer CA 2012 The molecular basis of drug resistance against hepatitis C virus NS3/4A protease inhibitors. PLoS Pathog 8:e1002832. doi:10.1371/journal.ppat.1002832. 22910833 PMC3406087

[11] Pawlotsky JM 2013 NS5A inhibitors in the treatment of hepatitis C. J Hepatol 59:375–382. doi:10.1016/j.jhep.2013.03.030. 23567084

[12] Gao M 2013 Antiviral activity and resistance of HCV NS5A replication complex inhibitors. Curr Opin Virol 3:514–520. doi:10.1016/j.coviro.2013.06.014. 23896281

[13] Jensen SB, Serre SB, Humes DG, Ramirez S, Li YP, Bukh J, Gottwein JM 2015 Substitutions at NS3 residue 155, 156, or 168 of hepatitis C virus genotypes 2 to 6 induce complex patterns of protease inhibitor resistance. Antimicrob Agents Chemother 59:7426–7436. doi:10.1128/AAC.01953-15. 26392503 PMC4649233

[14] Pawlotsky JM 2016 Hepatitis C virus resistance to direct-acting antiviral drugs in interferon-free regimens. Gastroenterology 151:70–86. doi:10.1053/j.gastro.2016.04.003. 27080301

[15] Lahser FC, Bystol K, Curry S, McMonagle P, Xia E, Ingravallo P, Chase R, Liu R, Black T, Hazuda D, Howe AY, Asante-Appiah E 2016 The combination of grazoprevir, a hepatitis C virus (HCV) NS3/4A protease inhibitor, and elbasvir, an HCV NS5A inhibitor, demonstrates a high genetic barrier to resistance in HCV genotype 1a replicons. Antimicrob Agents Chemother 60:2954–2964. doi:10.1128/AAC.00051-16. 26926625 PMC4862497

[16] Falade-Nwulia O, Suarez-Cuervo C, Nelson DR, Fried MW, Segal JB, Sulkowski MS 2017 Oral direct-acting agent therapy for hepatitis C virus infection: a systematic review. Ann Intern Med 166:637–648. doi:10.7326/M16-2575. 28319996 PMC5486987

[17] American Association for the Study of Liver Diseases and Infectious Diseases Society of America 2017 Hepatitis C guidance: recommendations for testing, managing, and treating hepatitis C virus. American Association for the Study of Liver Diseases, Alexandria, VA, and Infectious Diseases Society of America, Arlington, VA http://www.hcvguidelines.org. 10.1002/hep.31060 PMC9710295 31816111

[18] European Association for the Study of the Liver 2017 EASL recommendations on treatment of hepatitis C. J Hepatol 66:153–194. doi:10.1016/j.jhep.2016.09.001. 36464532

[19] Merck & Co., Inc 2016 Zepatier (elbasvir and grazoprevir) tablets, for oral use package insert. Merck & Co., Inc., Whitehouse Station, NJ.

[20] Jacobson IM, Asante-Appiah E, Wong P, Black T, Howe A, Wahl J, Robertson MN, Nguyen B-Y, Shaughnessy M, Hwang P, Barr E, Hazuda D 2015 Prevalence and impact of baseline NS5A resistance-associated variants (RAVs) on the efficacy of elbasvir/grazoprevir (EBR/GZR) against GT1a infection. Hepatology 62:1393A–1394A.

[21] McPhee F, Hernandez D, Yu F, Ueland J, Monikowski A, Carifa A, Falk P, Wang C, Fridell R, Eley T, Zhou N, Gardiner D 2013 Resistance analysis of hepatitis C virus genotype 1 prior treatment null responders receiving daclatasvir and asunaprevir. Hepatology 58:902–911. doi:10.1002/hep.26388. 23504694

[22] Janssen Products. 2013 Olysio (simeprevir). Highlights of prescribing information. Janssen Therapeutics, Titusville, NJ.

[23] Hernandez D, Zhou N, Ueland J, Monikowski A, McPhee F 2013 Natural prevalence of NS5A polymorphisms in subjects infected with hepatitis C virus genotype 3 and their effects on the antiviral activity of NS5A inhibitors. J Clin Virol 57:13–18. doi:10.1016/j.jcv.2012.12.020. 23384816

[24] Lontok E, Harrington P, Howe A, Kieffer T, Lennerstrand J, Lenz O, McPhee F, Mo H, Parkin N, Pilot-Matias T, Miller V 2015 Hepatitis C virus drug resistance-associated substitutions: state of the art summary. Hepatology 62:1623–1632. doi:10.1002/hep.27934. 26095927

[25] Krishnan P, Schnell G, Tripathi R, Ng T, Reisch T, Beyer J, Dekhtyar T, Irvin M, Xie W, Larsen L, Mensa F, Pilot-Matias T, Collins C 2017 Pooled resistance analysis in HCV genotype 1-6-infected patients treated with glecaprevir/pibrentasvir in phase 2 and 3 clinical trials. J Hepatol 66(Suppl 1):S500. 10.1128/AAC.01249-18 PMC6153825 30061289

[26] Puoti M, Foster G, Wang S, Mutimer D, Gane E, Moreno C, Chang TT, Lee SS, Marinho R, Dufour JF, Pol S, Hezode C, Gordon SC, Strasser SI, Thuluvath PJ, Liu R, Pilot-Matias T, Mensa F 2017 High SVR rates with eight and twelve weeks of pangenotypic glecaprevir/pibrentasvir: integrated efficacy and safety analysis of genotype 1-6 patients without cirrhosis. J Hepatol 66(Suppl 1):S721.

[27] Liu R, Curry S, McMonagle P, Yeh WW, Ludmerer SW, Jumes PA, Marshall WL, Kong S, Ingravallo P, Black S, Pak I, DiNubile MJ, Howe AY 2015 Susceptibilities of genotype 1a, 1b, and 3 hepatitis C virus variants to the NS5A inhibitor elbasvir. Antimicrob Agents Chemother 59:6922–6929. doi:10.1128/AAC.01390-15. 26303801 PMC4604396

[28] Pilot-Matias T, Tripathi R, Cohen D, Gaultier I, Dekhtyar T, Lu L, Reisch T, Irvin M, Hopkins T, Pithawalla R, Middleton T, Ng T, McDaniel K, Or YS, Menon R, Kempf D, Molla A, Collins C 2015 *In vitro* and *in vivo* antiviral activity and resistance profile of the hepatitis C virus NS3/4A protease inhibitor ABT-450. Antimicrob Agents Chemother 59:988–997. doi:10.1128/AAC.04227-14. 25451053 PMC4335891

[29] Chase R, Black S, McMonagle P, Guo Z, Curry S, Nachbar R, Howe AYM, Howe JA 2013 Characterization of resistance associated variants selected in GT1, GT2, and GT3 replicons by the HCV NS3/4A protease inhibitor MK5172, abstr 25. Abstr Int Conf Viral Hepatitis, New York, NY.

[30] Summa V, Ludmerer SW, McCauley JA, Fandozzi C, Burlein C, Claudio G, Coleman PJ, Dimuzio JM, Ferrara M, Di Filippo M, Gates AT, Graham DJ, Harper S, Hazuda DJ, Huang Q, McHale C, Monteagudo E, Pucci V, Rowley M, Rudd MT, Soriano A, Stahlhut MW, Vacca JP, Olsen DB, Liverton NJ, Carroll SS 2012 MK-5172, a selective inhibitor of hepatitis C virus NS3/4a protease with broad activity across genotypes and resistant variants. Antimicrob Agents Chemother 56:4161–4167. doi:10.1128/AAC.00324-12. 22615282 PMC3421554

[31] Schnell G, Tripathi R, Beyer J, Reisch T, Krishnan P, Lu L, Dekhtyar T, Hall C, Vilchez RA, Pilot-Matias T, Collins C 2015 Hepatitis C virus genotype 4 resistance and subtype demographic characterization of patients treated with ombitasvir plus paritaprevir/ritonavir. Antimicrob Agents Chemother 59:6807–6815. doi:10.1128/AAC.01229-15. 26282418 PMC4604390

[32] Taylor JG, Appleby T, Barauskas O, Chen X, Dvory-Sobol H, Gong R, Lee J, Nejati E, Schultz BE, Wang Y, Yang C, Yu M, Zipfel S, Chan K 2015 Preclinical profile of the pan-genotypic HCV NS3/4A protease inhibitor GS-9857. J Hepatol 62(Suppl 2):S681.

[33] Ng TI, Krishnan P, Pilot-Matias T, Kati W, Schnell G, Beyer J, Reisch T, Lu L, Dekhtyar T, Irvin M, Tripathi R, Maring C, Randolph JT, Wagner R, Collins C 2017 *In vitro* antiviral activity and resistance profile of the next-generation hepatitis C virus NS5A inhibitor pibrentasvir. Antimicrob Agents Chemother 61:e02558-16. doi:10.1128/AAC.02558-16. 28193664 PMC5404558

[34] Prichard MN, Aseltine KR, Shipman C Jr 1993 MacSynergy II, v1.0. University of Michigan, Ann Arbor, MI.

[35] Foster GR, Gane E, Asatryan A, Asselah T, Ruane PJ, Pol S, Poordad F, Stedman CA, Dore G, Roberts SK, Kaita K, Vierling J, Vargas HE, Kort J, Lin CW, Liu R, Ng T, Mensa F 2017 ENDURANCE-3: safety and efficacy of glecaprevir/pibrentasvir compared to sofosbuvir plus daclatasvir in treatment-naïve HCV genotype 3-infected patients without cirrhosis. J Hepatol 66(Suppl 1):S33.

[36] Chen ZW, Li H, Ren H, Hu P 2016 Global prevalence of pre-existing HCV variants resistant to direct-acting antiviral agents (DAAs): mining the GenBank HCV genome data. Sci Rep 6:20310. doi:10.1038/srep20310. 26842909 PMC4740856

[37] Berger KL, Triki I, Cartier M, Marquis M, Massariol MJ, Böcher WO, Datsenko Y, Steinmann G, Scherer J, Stern JO, Kukolj G 2014 Baseline hepatitis C virus (HCV) NS3 polymorphisms and their impact on treatment response in clinical studies of the HCV NS3 protease inhibitor faldaprevir. Antimicrob Agents Chemother 58:698–705. doi:10.1128/AAC.01976-13. 24217701 PMC3910880

[38] Shepherd SJ, Abdelrahman T, MacLean AR, Thomson EC, Aitken C, Gunson RN 2015 Prevalence of HCV NS3 pre-treatment resistance associated amino acid variants within a Scottish cohort. J Clin Virol 65:50–53. doi:10.1016/j.jcv.2015.02.005. 25766988 PMC4728298

[39] Jimenez-Sousa MÁ Gutiérrez-Rivas M, Álvaro-Meca A, García-Álvarez M, Harrigan PR, Fedele CG, Briz V, Vázquez-Morón S, Resino S 2016 NS3 resistance-associated variants (RAVs) in patients infected with HCV genotype 1a in Spain. PLoS One 11:e0163197. doi:10.1371/journal.pone.0163197. 27685471 PMC5042525

[40] Palanisamy N, Danielsson A, Kokkula C, Yin H, Bondeson K, Wesslén L, Duberg AS, Lennerstrand J 2013 Implications of baseline polymorphisms for potential resistance to NS3 protease inhibitors in hepatitis C virus genotypes 1a, 2b and3a. Antiviral Res 99:12–17. doi:10.1016/j.antiviral.2013.04.018. 23648709

[41] U.S. Food and Drug Administration, Center for Drug Evaluation and Research 2016 Application number 208261Orig1s000, Zepatier microbiology/virology review. U.S. Food and Drug Administration, Center for Drug Evaluation and Research, Rockville, MD https://www.accessdata.fda.gov/drugsatfda_docs/nda/2016/208261Orig1s000MicroR.pdf Accessed 6 July 2017.

[42] Asselah T, Kowdley KV, Zadeikis N, Wang S, Hassanein T, Horsmans Y, Colombo M, Calinas F, Aguilar H, de Ledinghen V, Mantry PS, Hezode C, Marinho RT, Agarwal K, Nevens F, Elkhashab M, Kort J, Liu R, Ng TI, Krishnan P, Lin CW, Mensa FJ 22 9 2017 Efficacy of glecaprevir/pibrentasvir for 8 or 12 weeks in patients with HCV genotype 2, 4, 5, or 6 infection without cirrhosis. Clin Gastroenterol Hepatol doi:10.1016/j.cgh.2017.09.027. 28951228

[43] Lawitz EJ, O'Riordan WD, Asatryan A, Freilich BL, Box TD, Overcash JS, Lovell S, Ng TI, Liu W, Campbell A, Lin CW, Yao B, Kort J 2015 Potent antiviral activities of the direct-acting antivirals ABT-493 and ABT-530 with three-day monotherapy for hepatitis C virus genotype 1 infection. Antimicrob Agents Chemother 60:1546–1555. doi:10.1128/AAC.02264-15. 26711747 PMC4775945

[44] Poordad F, Felizarta F, Asatryan A, Sulkowski MS, Reindollar RW, Landis CS, Gordon SC, Flamm SL, Fried MW, Bernstein DE, Lin CW, Liu R, Lovell SS, Ng TI, Kort J, Mensa FJ 2017 Glecaprevir and pibrentasvir for 12 weeks for hepatitis C virus genotype 1 infection and prior direct-acting antiviral treatment. Hepatology 66:389–397. doi:10.1002/hep.29081. 28128852 PMC5573922

[45] Zeuzem S, Feld J, Wang S, Bourliere M, Wedemeyer H, Gane E, Flisiak R, Chuang WL, Flamm S, Kwo P, Sepulveda-Arzola G, Soto-Malave R, Puoti M, Tam E, Bruck R, Fuster F, Paik SW, Felizarta F, Fu B, Ng TI, Lin CW, Mensa F 2016 ENDURANCE-1: a phase 3 evaluation of the efficacy and safety of 8- versus 12-week treatment with glecaprevir/pibrentasvir (formerly ABT-493/ABT-530) in HCV genotype 1 infected patients with or without HIV-1 co-infection and without cirrhosis. Hepatology 64(Suppl 1):132A–133A.

[46] Forns X, Lee SS, Valdes J, Lens S, Ghalib R, Aguilar H, Felizarta F, Hassanein T, Hinrichsen H, Rincon D, Morillas R, Zeuzem S, Horsmans Y, Nelson DR, Yu Y, Krishnan P, Lin CW, Kort JJ, Mensa FJ 2017 Glecaprevir plus pibrentasvir for chronic hepatitis C virus genotype 1, 2, 4, 5, or 6 infection in adults with compensated cirrhosis (EXPEDITION-1): a single-arm, open-label, multicentre phase 3 trial. Lancet Infect Dis 10:1062–1068. doi:10.1016/S1473-3099(17)30496-6. 28818546

[47] Gane E, Lawitz E, Pugatch D, Papatheodoridis G, Bräu N, Brown A, Pol S, Leroy V, Persico M, Moreno C, Colombo M, Yoshida EM, Nelson DR, Lei Y, Kosloski M, Mensa F 2016 EXPEDITION-4: efficacy and safety of glecaprevir/pibrentasvir (ABT-493/ABT-530) in patients with renal impairment and chronic hepatitis C virus genotype 1-6 infection. Hepatology 64(Suppl 1):1125A.

[48] Bukh J, Apgar CL, Engle R, Govindarajan S, Hegerich PA, Tellier R, Wong DC, Elkins R, Kew MC 1998 Experimental infection of chimpanzees with hepatitis C virus of genotype 5a: genetic analysis of the virus and generation of a standardized challenge pool. J Infect Dis 178:1193–1197. doi:10.1086/515683. 9806059

[49] Konstantinidis AK, Richardson PL, Kurtz KA, Tripathi R, Chen CM, Huang P, Randolph J, Towne D, Donnelly J, Warrior U, Middleton T, Kati WM 2007 Longer wavelength fluorescence resonance energy transfer depsipeptide substrates for hepatitis C virus NS3 protease. Anal Biochem 368:156–167. doi:10.1016/j.ab.2007.06.020. 17644059

[50] Lohmann V, Korner F, Koch J, Herian U, Theilmann L, Bartenschlager R 1999 Replication of subgenomic hepatitis C virus RNAs in a hepatoma cell line. Science 285:110–113. doi:10.1126/science.285.5424.110. 10390360

[51] Prichard MN, Shipman C Jr 1996 Analysis of combinations of antiviral drugs and design of effective multidrug therapies. Antivir Ther 1:9–20. 11322261

[52] Prichard MN, Prichard LE, Shipman C Jr 1993 Strategic design and three-dimensional analysis of antiviral drug combinations. Antimicrob Agents Chemother 37:540–545. doi:10.1128/AAC.37.3.540. 8384816 PMC187704

